# OSMR gene effect on the pathogenesis of chronic autoimmune Urticaria via the JAK/STAT3 pathway

**DOI:** 10.1186/s10020-018-0025-6

**Published:** 2018-06-05

**Authors:** Xiao-Yan Luo, Qun Liu, Huan Yang, Qi Tan, Li-Qiang Gan, Fa-Liang Ren, Hua Wang

**Affiliations:** 1 Department of Dermatology, Children’s Hospital of Chongqing Medical University, Chongqing, 400014 China; 2 Ministry of Education Key Laboratory of Child Development and Disorders, Chongqing, 400014 China; 3 The Division of Allergy and Clinical Immunology, Johns Hopkins University School of Medicine, Baltimore, MD 21224 USA; 4 China International Science and Technology Cooperation Base of Child Development and Critical Disorders, Chongqing, 400014 China; 5 Chongqing Key Laboratory of Pediatrics, No.136, Zhongshan Er Road, Yuzhong District, Chongqing, 400014 China

**Keywords:** OSMR gene, JAK/STAT3 signaling pathway, Chronic autoimmune urticaria, Pathogenesis, Autoimmunity

## Abstract

**Background:**

Chronic autoimmune urticaria (CAU) is a common skin disease and remains unclear understanding of pathogenesis in the vast majority of cases. In order to explore a new therapy for CAU, the current study was performed to investigate the possible functioning of the Oncostatin M receptor (OSMR) gene in the autoimmunity of CAU via regulation of the JAK/STAT3 signaling pathway.

**Methods:**

CAU skin tissues from 24 CAU patients and normal skin tissues from normal subjects were collected. Hematoxylin-eosin (HE) staining was conducted to count eosinophils, and immunohistochemistry was carried out to detect the positive rate of OSMR expression in two kinds of skin tissues. A total of 72 Kunming (KM) mice were selected, and 60 mice were used for establishing CAU models and later transfected with different plasmids. The expression of inflammatory factors was evaluated by enzyme-linked immunosorbent assays (ELISA). Expressions of janus kinase (JAK), signal transducer and activator of transcription 3 (STAT3), interferon-stimulated gene 15 (ISG15), CT10-regulated kinase (CRK), and interferon regulatory factor 9 (IRF9) were identified using Western blot assay and reverse transcription quantitative polymerase chain reaction (RT-qPCR). Epithelial cell proliferation was assessed by 3-[4,5-dimethylthiazol-2-yl]-2,5-diphenyl tetrazolium bromide (MTT) assay, and cell cycle distribution and cell apoptosis were assessed using flow cytometry.

**Results:**

The findings confirm that OSMR protein expression and histamine release rate are highly elevated in human CAU skin tissues, and the expression of the JAK/STAT3 signaling pathway-related genes (OSMR, JAK2, STAT3, ISG15, CRK and IRF9) was up-regulated. OSMR gene silencing in CAU mice significantly decreases the content of inflammatory factors (IL-1, IL-6, IFN-γ, and IgE), the number of eosinophils, and reduces the expression of the JAK/STAT3 signaling pathway related genes, and further enhances cell proliferation, promotes cell cycle entry and inhibits apoptosis of epithelial cells.

**Conclusion:**

All aforementioned results indicate that OSMR gene silencing inhibits the activation of the JAK/STAT3 signaling pathway, thereby suppressing the development of CAU.

## Background

Chronic urticaria (CU), an immune-mediated inflammatory disease, is defined as the spontaneous or inducible appearance of hives, angioedema or both lasting at least 6 weeks and presenting with numerous subtypes, all greatly damage patients’ quality of life (Bingham 3rd, 2008; Gimenez-Arnau et al., 2015). CU, the potentially debilitating skin condition, is known to affect up to 1% of the general population with different durations, usually several months, but occasionally decades (Ventura et al., 2013). Chronic idiopathic urticaria (CIU) is a common type of CU accounting for over 70% cases of CU, and chronic autoimmune urticaria (CAU), a subgroup of CIU, accounts for more than 30% of CIU. CIU is characterized by severe and persistent wheals accompanied by redness and itching (Goh & Tan, 2009; Abd El-Azim & Abd, 2011). CAU is caused by anti-FcepsilonRI and less normally, by anti-IgE autoantibodies that result in the activation of mast cells and basophils (Goh & Tan, 2009). Currently, clinical suspicion and autologous serum skin test (ASST) are regarded as the basis of CAU diagnosis (Abd El-Azim & Abd, 2011). Previously, the role of omalizumab in treating refractory CAU patients was studied, and proven possible (Al-Ahmad, 2010). In addition, mizoribine was found to be an effective therapy in some CAU patients, and may possibly be effective for patients not responsive to traditional therapy (Hashimoto et al., 2012). CAU patients are poor responders to antihistamine therapy, which leads to the necessity of immunosuppressive therapy (Cherrez Ojeda et al., 2009). Therefore, new genetic methods are required to find the possible ways for the treatment of CAU.

Oncostatin M (OSM), a member of the interleukin 6 (IL-6) family of cytokines, plays important roles in various biological functions, including inflammatory responses and metabolic diseases (Komori et al., 2013). OSM secreted by skin-infiltrating T-lymphocytes is considered to be a potential keratinocyte activator correlated to skin inflammation (Boniface et al., 2007). Oncostatin M receptor (OSMR) gene is located at 5p13.1, and can bind to gp130 to mediate the biological functions of OSM (Hong et al., 2011; Deng et al., 2009). Therapies based on OSMR have been reported for treatment of various cancers including cervical squamous cell carcinoma and lung adenocarcinomas, as well as skin diseases such as familial primary localized cutaneous amyloidosis (Caffarel & Coleman, 2014; Chen et al., 2008; Arita et al., 2008). Janus kinase-signal transducer and activator of transcription (JAK/STAT) transmits information received from extracellular polypeptide signals through transmembrane receptors, directly to the target gene promoters in the nucleus, providing a mechanism for regulation of transcriptional without second messengers (Aaronson & Horvath, 2002). JAKs are required for numerous inflammatory cytokine signaling pathways, and are implicated in the pathogenesis of chronic dermatitis, atopic dermatitis and psoriasis, and JAK inhibitors are thus promising therapeutic candidates for chronic dermatitis (Tanimoto et al., 2018). Additionally, JAK inhibitors, which are also used to inhibit cytokine signaling, are assumed to be a possible mean of treating skin inflammatory disorders such as contact dermatitis (Amano et al., 2016). It has been reported that OSM is released in inflammatory conditions, and it signals primarily via the JAK/STAT pathway by combining with its receptor complex (Hermanns, 2015). The heterodimeric receptor complex combined with gp130 and OSMR could activate a signaling pathway involved in JAKs as well as transcription factors of the STAT family (Hintzen et al., 2008). However, further verification is required in order to explore whether the OSMR gene is involved in the pathogenesis of CAU through the JAK/STAT3 signaling pathway. Therefore, the current study aims to explore the role of OSMR gene silencing in the pathogenesis of CAU and its underlying mechanism involving the JAK/STAT3 signaling pathway.

## Methods

### Ethics statement

This study was approved by the Ethics Committee of Children’s Hospital of Chongqing Medical University, and signed informed consents were obtained from all patients/guardians. In addition, the experiments were in accordance with the ethical standards, and all efforts were made to minimize the suffering of the animals included in the study.

### Study subjects

CAU skin tissues from 24 CAU patients of Children’s Hospital of Chongqing Medical University were collected, and normal skin tissues from skin grafts of 24 plastic surgery patients were selected as controls. Skin biopsy specimens were rapidly frozen in liquid nitrogen in order to prevent protein denaturation until total RNA was extracted from the specimens. The 24 CAU patients included 11 males and 13 females, with a mean age of 10 years. The average courses of disease of patients were 6.67 months (range 2–16 months). All patients had been treated with antihistaminic agents, and some patients underwent treatment with corticosteroids but with poor efficacy, for 16 of them complained of joint pain, gastrointestinal or respiratory symptoms.

### Hematoxylin-eosin (HE) staining

Skin tissues extracted from CAU patients were fixed in 4% paraformaldehyde for 24 h, washed, dehydrated, cleared, waxed, embedded, sectioned, and made into paraffin sections. After that, the sections were stained with hematoxylin for observing the eosinophil infiltration in skin tissue of patients.

### Immunohistochemistry

The sections underwent routine dewaxing, dehydration with gradient ethanol, antigen-repair under high pressure for 1.5 min, and cooling under tap water for 10 min. After the remaining tap water on the sections was removed under running water, the sections were added one drop of endogenous peroxidase blocking solution, incubated at room temperature for 10 min, and rinsed with phosphate buffer solution (PBS) (3 min, 3 times). Then, the sections were added with suitable amount of primary antibody, namely rabbit anti human immunoglobulin G (IgG) antibody for incubation overnight at 4 °C, followed by rinsing with PBS after being taken out (3 min, 3 times), and incubation with the biotin-labeled secondary mouse anti rabbit monoclonal antibody IgG/horseradish peroxidase (HRP) (dilution ratio of 1: 1000, ab6759, Abcam, Inc., Cambridge, MA, USA) at 37 °C for 30 min. After incubation with the two types of antibodies, the sections were rinsed with PBS (3 min, 3 times), dealt by streptomyces anti biotin catalase complex for 15 min, colored with 3,3′-diaminobenzidine (DAB), and then rinsed under tap water to terminate the whole reaction. Later, the sections were re-stained with hematoxylin, dehydrated, cleared, and mounted. PBS was used as the negative control instead of the primary antibody. Finally, each section was randomly photographed under a light microscope (at 10× & 40× magnification) to get 5 non-overlapping visual fields. At last, 100 cells were counted in each visual field randomly, and the percentage of positive cells = positive cells/total cells.

### CAU animal model establishment

A total of 72 Kunming (KM) mice weighing 18~ 25 g (J018, Better Biotechnology Co., Ltd., Nanjing, China) were selected for the study, amongst which 60 mice were used for the establishment of CAU mouse models, and the other 12 untreated mice were regarded as the normal group. Each intraplantar of mice was treated with 0.05 mL 5% physiological saline solution containing ovalbumin (the total amount of injection for a mouse was 0.1 mL). Meanwhile, each mouse received pertussis vaccines (4 × 10^9^ U) via intraperitoneal injections. After 12–14 d, the mice were sacrificed by using the neck-breaking method and blood was drawn. Mice blood was collected and centrifuged in order to separate the antiserum, which was stored in a refrigerator for later use (mixed antisera was selected from 5 sensitized mice). With the addition of normal saline (dilution ratio of 1: 10), 0.03 m^1^L antiserum was injected into the abdominal wall of the mice. Subsequently, antigen attack was conducted by injections of 1 mL normal saline (containing 1 mg ovalbumin). The indications of pruritus include systemic pruritus-head scratching by paw, torso scratching by hind claws, and biting all parts of the body by mouth. The number and total duration of pruritus in each mouse was recorded within 30 min of the 1 mL dextran injections through the tail vein. The CAU mouse model was considered to be successfully established if the number and total duration of pruritus were significantly higher than the normal mice (Yagami et al., 2017). A total of 60 mice were successfully established as CAU models which were classified into: the blank group (model mice without any treatment), the negative control group (NC) (model mice transfected with empty vector plasmid), the OSMR-siRNA group (model mice transfected with OSMR-siRNA plasmid), the anti-phospho-STAT3 (Tyr705) + OSMR-siRNA group (model mice transfected with OSMR-siRNA plasmid + the JAK/STAT3 signaling pathway agonist), and the Tyr705 group (model mice treated with the JAK/STAT3 signaling pathway agonist) groups. The plasmids used in the experiments were purchased from Vigene Biotechnology Co., Ltd. (Shandong, China). Then, attention was paid to the number and total duration of pruritus within 30 min. The mice were sacrificed after successful transfection with corresponding plasmids. Then, mice skin specimens with wheal or rash were extracted and stored at − 80 °C. Eosinophil counting was conducted by routine method and the absolute value was recorded.

### Isolation and culture of mast cells

The foreskin of children (1–9 years) was extracted by circumcision under aseptic conditions. The skin grafts were incubated in a RPMI 1640 culture medium (containing 100 U/mL penicillin and 100 μg/mL streptomycin) after blood was removed using normal saline. Subcutaneous tissues were isolated and rinsed with modified Tyrode solution (containing 137 mmol/L NaCl, 2.7 mmol/L KCl, 0.4 mmol/L NaH_2_PO_4_, 5.6 mmol/L Glucose, 10 mmol/L HEPES, 1 mmol/L CaCl and 1 mmol/L MgCl_2_). Subsequently, the skin grafts were cut sliced tissue fragments of 1 mm^2^, and placed in RPMI 1640 culture medium containing 1.5 mg/mL type I collagenase and 0.5 mg/mL hyaluronidase for 4-h culturing in an incubator containing 5% CO_2_ in air and saturated humidity at 37 °C. Next, the fragments were isolated in order to a form a cell suspension by repeated blowing and beating of digestive juice with a straw. The suspension was filtrated with a stainless steel filter net. After removal of the tissue fragments and larger cell clusters, the filtrate was collected, rinsed with icy Tyrode solution, re-suspended in a RPMI 1640 culture medium containing 10% fetal bovine serum (FBS), 100 U/mL penicillin and 100 μg/mL streptomycin, and cultured in a 5% CO_2_ incubator with saturated humidity at 37 °C for 12 h. Subsequently, the culture medium was collected after gently shaking the culture bottle.

### Histamine release test and enzyme linked immunosorbent assay (ELISA)

A total of 75 μL serum samples and normal serum samples obtained from CAU patients and healthy individuals were respectively mixed with 75 μL mast cell suspension, and incubated at 37 °C for 20 min, followed by centrifugation at 1610×g for 15 min, and the supernatant was collected which was used for evaluation of histamine release rate (the compound tube was used for aforementioned evaluation). The histamine release rate in CAU patients and normal individuals was determined by ELISA. Firstly, the test sample and reagent kit (ZK-G7274, Zike Biotechnology Co., Ltd., Shenzhen, China) were subjected to acylation and dilution following the standard and quality control in accordance with the operating procedures. Then, 50 μL test serum samples were obtained from CAU patients and healthy individuals. A total of 50 μL polyclonal anti-histamine antibody, enzyme conjugates and histamine antiserum were added into each well successively, which were mixed evenly and incubated at room temperature for 3 h, followed by plate rinsing five times. After that, the freshly prepared 200 μL 3,3′,5,5′-tetramethylbenzidine (TMB) substrate solution was added into each well for further incubation at room temperature for 20 min, followed by plate rinsing five times, and pat to dry the plate. Later, 100 μL TMB stop solution was added to each well to terminate the reaction, and the absorbance (A) value measured using a microplate reader at the excitation wavelength of 450 nm. Content of histamine in samples was calculated using a standard curve, and the formula was as follows: X = A × 50/m. The histamine spontaneous release rate = histamine spontaneous release/total histamine content × 100%, and histamine release rate of samples = histamine content/total histamine content × 100%. Interleukin-1 (IL-1) ELISA kit (SBJ-M0582), IL-6 ELISA kit (SBJ-M0044), interferon (IFN)-γ ELISA kit (SBJ-M0038), IgE ELISA kit (SBJ-M0499) were used to measure the contents of IL-1, IL-6, IFN-γ and IgE according to the protocols provided by the manufacturer. All the kits were purchased from Nanjing SenBeiJia Biological Technology Co., Ltd. (Nanjing, Jiangsu, China).

### Reverse transcription quantitative polymerase chain reaction (RT-qPCR)

Skin tissues (100 mg) were collected from mice in each group, placed into a glass grinder and added with 1 mL tissue lysate (BB-3209, Bestbio Technology, Co., Ltd., Shanghai, China), ground to an even homogenate by ice-bath, and placed on a nucleic acid protein analyzer (BioPhotometer D30, Eppendorf, Hamburg, Germany) for the detection of absorbance ratio and RNA concentration. The results of optical density (OD) value at 260 nm/ that at 280 nm placed between 1.8~ 2.0 is indicative of highly purified RNA. Total RNA were extracted from 100 mg skin tissues in each group using the Trizol reagent (16,096,020, Invitrogen Inc., Carlsbad, CA, USA) in accordance with the instructions of the manufacturer, and PrimeScript RT Reagent kit (Fermentas, Maryland, NY, USA) was performed for RNA reserve transcription into cDNA. The reserve transcription conditions were as follows: 70 °C for 5 min, ice-bathing for 3 min, 37 °C for 60 min, and 95 °C for 10 min. The cDNA was temporarily preserved at − 20 °C in a refrigerator. Primers of OSMR, JAK2, STAT3, ISG15, CRK, IRF9, and GAPDH were synthesized by Takara (Takara Biotechnology Co., Ltd., Liaoning China). PCR amplification was performed to the target genes with 25 μL reaction system as follows: 300 ng cDNA, 1× PCR buffer solution, 200 μmol/L dNTPs, 80 pmol/L forward and reverse primers, 0.5 U Taq enzyme (S10118, Yuanye Biotechnology Co., Ltd., Shanghai, China) with the reaction system of pre-denaturation at 94 °C for 5 min, denaturation at 94 °C for 30 s, annealing at 54 °C for 30 s, extension at 72 °C for 30 s, all cycles were repeated 30 times with the last reaction at 72 °C for 10 and preserved at 4 °C. The primer sequences of OSMR, janus kinase 2 (JAK2), signal transducer and activator of transcription 3 (STAT3), interferon-stimulated gene 15 (ISG15), CT10-regulated kinase (CRK), and interferon regulatory factor 9 (IRF9), and glyceraldehyde-3-phosphate dehydrogenase (GAPDH) are shown in Table 1, and GAPDH was regarded as the internal control. The relative ratio of genes between experimental group and control group were calculated using the 2^-ΔΔCt^ method with the formula as: ΔΔCT = ΔC_texperimental group_ - ΔCt_control group_, among which ΔCt = Ct_OSMR_–Ct_GAPDH_ (Denley et al., 2013). Ct is the amplification cycle number when real time fluorescence intensity reached the set threshold. At such time, the amplification was in logarithmic phase of growth and the experiment was performed in triplicate.

**Table 1:** RT-qPCR primer sequences

Genes	Sequences
OSMR	F: 5’-AGAAACTGGCACACCATCCT-3′
	R: 5’-ACTGCCCTAATGACCAGTGC-3′
STAT3	F: 5’-GCCACGTTGGTGTTTCATAATC-3′
	R: 5’-TTCGAAGGTTGTGCTGATAGAG-3′
JAK2	F: 5’-TGCTGTCCAGACAAGAATGC-3′
	R: 5’-TCCTTCTCTGCCAACGTCTT-3′
ISG15	F: 5’-CACAGTCCTGCTGGTGG-3′
	R: 5’-GGCGATACTGCGACCCT − 3′
CRK	F: 5’-GGCAGGGTAGTGGAGTGAT-3′
	R: 5’-AGGCTGTCTTGTCGTAGGC-3′
IRF9	F: 5’-TGCTTCCTCCAGAGCCAGAC-3′
	R: 5’-CACAAGGCGGCAATCCAG-3’
GAPDH	F: CCACCCATGGCAAATTCCATGGCA
	R: TCTAGACGGCAGGTCAGGTCCAC

*RT-qPCR* reverse transcription quantitative polymerase chain reaction, *OSMR* oncostatin M receptor, *STAT3* signal transducer and activator of transcription 3, *JAK2* janus kinase 2, *ISG15* interferon-stimulated gene 15, *CRK* CT10-regulated kinase, *IRF9* interferon regulatory factor 9, *GAPDH* glyceraldehyde-3-phosphate dehydrogenase, *F* forward, *R* reverse

### Western blot analysis

Skin tissues (100 mg) of each group were extracted, placed in a glass grinder containing 1 mL tissue lysate (BB-3209, Bestbio Technology, Co., Ltd., Shanghai, China), and were ground to a homogenate by ice-bath, where after protein lysate was added in for tissue splitting at 4 °C for 30 min, centrifuged at 1610×g, 4 °C for 15 min, and the supernatant was collected. A bicinchoninic acid (BCA) kit (2020ES76, Yeasen Company, Shanghai, China) was employed in order to detect the concentration of each tissue samples. Firstly, deionized water was added to adjust the sample quantity of 30 μg protein lane. Then, 10% sodium dodecyl sulfate (SDS) separating glue and concentration glue was prepared. The sample tissues were mixed with the sample buffer, heated to a boil for 5 min, ice-bathed, centrifuged before being added into each lane with a micropipette for lectrophoretic separation. After that, the proteins on the membrane were transferred onto a nitrocellulose membrane (ZY-160FP, Zeye Biology, Shanghai, China), blocked with 5% skimmed power at 4 °C overnight. Later, diluted primary antibody, namely rabbit anti human polyclonal antibodies (dilution ratio of 1: 500), including OSMR (11226-R007, dilution ratio of 1: 400, Sino Biological Inc., Beijing, China), JAK2 (ab32101, dilution ratio of 1: 1000, Abcam Inc., Cambridge, MA, USA), STAT3 (ab68153, dilution ratio of 1: 1000, Abcam Inc., Cambridge, MA, USA), ISG15 (LS-C211809, dilution ratio of 1: 1000, Littleton, Colorado, USA), IRF9 (PAB28499, dilution ratio of 1: 400, Lianshuo Biological Technology, Wuhan, Hubei, China) and p-STAT3 (sc-56,747, Univ-bio, Shanghai, China) were added into the membrane for overnight incubation, followed by rinsing with PBS (5 mins, 3 times). The secondary antibody mouse anti rabbit IgG/HRP (Huabio Inc., Hangzhou, Zhejiang, China) was added for rocking incubation at 37 °C for 1 h, followed by rinsing with PBS at room temperature (5 mins, 3 times). At room temperature, the membrane was reacted with enhanced chemiluminescence (ECL) solution for 1 min, after which the membrane was amounted using cling-film with the liquid removed, and observed under an X-ray instrument (36209ES01, Qianchen Bioteachnology, Shanghai, China). GAPDH was regarded as the internal reference, and the grey value ratio of target band and GAPDH band was taken as the relative expression of sample protein. Each experiment was conducted three times.

### 3-(4,5-Dimethylthiazol-2-yl)-2,5-Diphenyltetrazolium bromide (MTT) assay

Skin tissues were extracted from mice in order to obtain keratinocytes after detachment, isolation and culture. The cells were allowed to reach around 80% confluence, and were rinsed with PBS two times, and detached by trypsin in order to prepare a single cell suspension. After counting, the cells were seeded into a 96-well plate at a density of 3 × 10^3^ ~ 6 × 10^3^ cells/well, with the cell volume in each well maintained to 0.2 mL. A total of 6 duplicate wells were set, and the cells were cultured in an incubator. At the 30 min, 1 h, 6 h, 12 h, 24 h, and 48 h time periods during the incubation, the culture plate was taken out and the original culture medium was replaced with 5 g/L 10% MTT solution (GD-Y1317, Guduo Biotechnology Co., Ltd., Shanghai, China) for further 4-h incubation. Later, 100 μL dimethyl sulphoxide (DMSO) (D5879-100ML, Sigma-Aldrich Chemical Company, St Louis, MO, USA) were added in, and gently oscillated for uniform mixing for 10 min. After formazan crystals produced by living cells were dissolved with DMSO, the cell plate was placed onto a microplate reader for detecting the OD value of each well at the excitation wavelength of 490 nm. The experiment was repeated three times and the time point was set as the abscissa and the OD value as the ordinate in order to plot the CAU cell activity graph.

### Scratch test

The mouse keratinocytes in the logarithmic phase of growth were selected, and isolated and cultured for 48 h. The cells were seeded in a 6-well plate at a density of 1 × 10^6^ cells in each well and cultured in a 5% CO_2_ incubator at 37 °C until cell confluence reached 95%. Then, a vertical linear scratch was drawn using a 20 μl micropipette, and then serum-free medium was added to the wells after the 6-well plate was washed with D-hanks solution. Sample cells were collected after scratching at 0 h and 36 h time periods with 3 visual fields at 100X magnification were photographed under a phase contrast microscope in order to compare the different scratch lanes. The healing rate of the scratch line was regarded as the cell migration and healing ability.

### Flow cytometry

The mouse keratinocytes were collected for detachment with 0.25% trypsin solution after isolation and culture for 48 h. The number of cell samples was adjusted to 1 × 10^6^ mL^− 1^. Then, 1 mL cells were centrifuged at 402×g for 10 min with the supernatant discarded and the cells collected. Per mL of the collected cells were added with 2 mL PBS before undergoing centrifugation. The supernatant was discarded, and the cells were fixed with 70% pre-cooled ethanol solution at 4 °C overnight. The following day, the fixed cells were rinsed with PBS two times, and a cell suspension of 100 μL (containing more than 10^6^ mL^− 1^) was selected, added with 1 mL 50 mg/L propidium iodide (PI) solution (containing RNAase) for 30-min incubation avoiding light exposure. After that, the cells were filtered with nylon net (300 mesh), and the cell cycle was analyzed using flow cytometry at an excitation wavelength of 488 nm.

Cell apoptosis was assessed using the Annexin V-fluorescein isothiocyanate (FITC)/PI double staining, and the cells were underwent the same process of cell cycle. The cells were cultured at 37 °C in an incubator containing 5% CO_2_ in air, and then were collected. After rinsing with PBS two times, the cells were centrifuged and re-suspended in 200 μL binding buffer, followed by the addition of fully-mixed 10 μL Annexin V-FITC and 5 μL PI for 15-min reaction avoiding light exposure at room temperature. Later, the cells were added with 300 μL binding buffer and placed onto the flow cytometry (6HT, Cellwar Bio-technology Co., Ltd., Wuhan, Hubei, China) for cell apoptosis detection at the excitation wavelength of 488 nm.

### Statistical analysis

Statistical analyses were performed using the SPSS 22.0 software (IBM Corp. Armonk, NY, USA). Measurement data were expressed as mean ± standard deviation. Differences between two groups were compared using the *t* test, and differences among multiple groups were analyzed using one-way analysis of variance (ANOVA). *p* < 0.05 was considered to be statistically significant.

## Results

### Elevated histamine releasing rate in CAU model mice signifies the successful model establishing

**Fig. 1:**
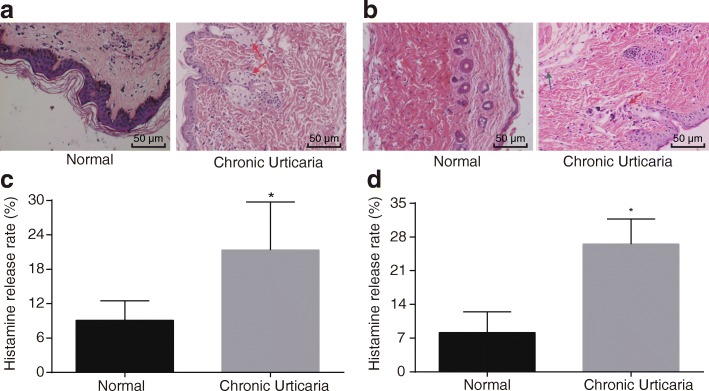
Deteriorated pathological changes and elevated histamine release rate in CAU revealed by HE staining and Histamine Release (× 200). Note: **a** normal skin tissues and CAU tissues in patients with CAU after HE staining, with the red arrows indicating towards the dermal vascular wall and the surrounding neutrophils and eosinophil infiltration (× 200); **b** normal skin tissues and CAU tissues in CAU mice after HE staining, with the red arrows indicating towards the dermal vascular wall and the surrounding neutrophils and eosinophil infiltration, and the blue arrow indicating towards the small amount of lymphocytic infiltration surrounding the dermal vascular wall (× 200); **c** histamine release experiment of human serum activated mast cells (*n* = 24); **d** histamine release experiment of mice serum activated mast cells in normal and CAU tissues (*n* = 12); *, *p* < 0.05 compared with the normal group; CAU, chronic autoimmune urticaria; HE, hematoxylin-eosin

In order to observe the histopathological changes of skin tissues after the occurrence of CAU, 24 CAU patients and 12 model mice (blank group) were recruited in the current study. As shown in Fig. 1a, all CAU specimens exhibited vasodilation, mild dermal edema and a perivascular or interstitial infiltrate composed of neutrophils, eosinophil and lymphocyte, while there were no telangiectasia and congestion in addition to lymphocytic and eosinophil infiltration around the dermal vessels in normal tissues. The observation results of skin tissues obtained from CAU mice were in accordance with the aforementioned findings (Fig. 1b).

According to results of the histamine releasing by mast cells, the release rates of histamine by normal human serum activated mast cells were all negative, while 10 positive cases and 14 negative cases of histamine release out of human CAU serum were observed with the release rate of (21.35 ± 8.40)% which was higher than the normal subjects (9.08 ± 3.42)% (*p* < 0.05) (Fig. 1c). In CAU model mice, CAU mice with no transfection had a histamine release rate of (26 ± 5.20)% compared with normal mice which was (8.16 ± 4.28)%, signifying a highly increased histamine release rate in CAU model mice and successful establishment of CAU models (Fig. 1d).

### Higher OSMR positive expression rate and elevated expression of the JAK/STAT3 signaling pathway-related genes in CAU skin tissues

**Fig. 2:**
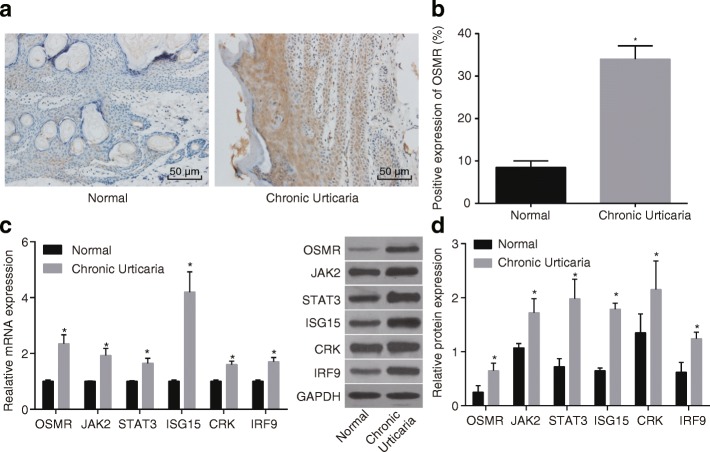
Increased protein and mRNA expressions of OSMR and JAK/STAT3 signaling pathway-related factors in skin tissues of CAU patients revealed by RT-qPCR assay and Western blot analysis (n = 24). Note: **a** OSMR protein expression in human CAU tissues and normal skin tissues under microscope (× 200); **b** comparison of OSMR protein expression rate in human CAU tissues and normal skin tissues revealed that OSMR protein expression was significantly higher in CAU tissues than the normal skin tissues; **c** mRNA expression of OSMR, JAK2, STAT3, ISG15, CRK and IRF9 was significantly higher in CAU tissues than those in normal skin tissues detected by RT-qPCR; **d** Western blot assay revealed increased protein expression of OSMR, JAK2, STAT3, ISG15, CRK and IRF9 in CAU tissues than those in normal skin tissues; *, *p* < 0.05 when compared with the normal skin tissues; CAU, chronic autoimmune urticaria; RT-qPCR, reverse transcription quantitative polymerase chain reaction; OSMR, Oncostatin M receptor; JAK2, janus kinase 2; STAT3, signal transducer and activator of transcription 3; ISG15, interferon-stimulated gene 15; CRK, CT10-regulated kinase; IRF9, interferon regulatory factor 9

In order to better investigate the expression of OSMR in CAU skin tissues, CAU and normal skin tissues were observed under the light microscope. The findings indicate that OSMR positive cells were primarily located in the superficial and middle dermis, surrounding the blood vessels and appendages in CAU skin tissues, with the positive granule largely located inside the epithelial cells. The OSMR positive expression rate in CAU skin tissues was 34.00%. However, a relatively small number of OSMR positive cells were observed in normal skin tissues with an OSMR positive expression rate of 8.50%, indicating that the OSMR positive expression rate in CAU skin tissues was significantly higher than normal skin tissues (*p* < 0.05) (Fig. 2a, b).

As OSMR plays a vital role in CAU skin tissues, RT-qPCR and Western blot assay were conducted in order to elucidate the relationship between OSMR and the JAK/STAT3 signaling pathway. The results (Fig. 2c) reveal that the mRNA expression of OSMR and the JAK/STAT3 signaling pathway-related genes, including JAK2, STAT3, ISG15, CRK and IRF9, were evidently elevated in CAU skin tissues (*p* < 0.05), and the same was confirmed by the Western blot analysis (*p* < 0.05) (Fig. 2d). All aforementioned findings indicate that CAU exhibits increased expression of OSMR and the activated JAK/STAT3 signaling pathway.

### Inhibited expression of CAU inflammatory factors as a result of OSMR silencing

**Fig. 3:**
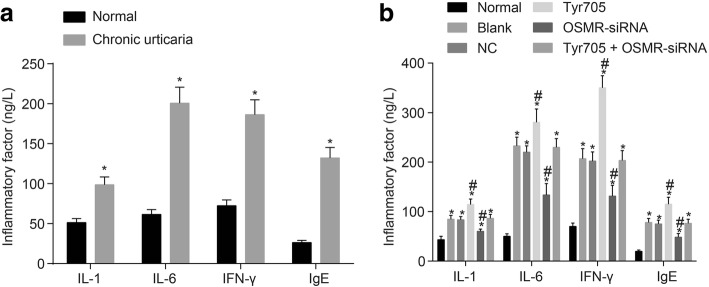
Decreased inflammatory factor content after transfection with OSMR-siRNA but increased by activation of the JAK/STAT3 signaling pathway. Notes: **a** inflammatory factor expression in serum of CAU patients (n = 24); **b** inflammatory factor expression in serum of CAU mouse tissues with different transfection (*n* = 12); *, *p* < 0.05 compared with the normal group; #, *p* < 0.05 compared with the blank group; CAU, chronic autoimmune urticaria; OSMR, Oncostatin M receptor; JAK, janus kinase; STAT, signal transducer and activator of transcription

The ELISA assay was employed to determine the levels of inflammatory factors such as IL-1, IL-6 and IFN-γ in CAU skin tissues. As shown in Fig. 3a, the inflammatory factors were found to be increased in CAU skin tissues. Mice models with different vectors transfection were established in order to testify the function of OSMR. Results of the ELISA assay (Fig. 3b) show that compared with the normal skin tissues, CAU tissues with no transfection and transfected with blank plasmids exhibited increased levels of IL-1, IL-6 and IFN-γ. Compared with CAU tissues with no transfection and transfected with blank plasmids, CAU tissues transfected with OSMR-siRNA exhibited decreased levels of IL-1, IL-6 and IFN-γ, with an opposite trend observed in CAU skin tissues transfected with Tyr705. Interestingly, there was no significant differences among CAU skin tissues with no transfection and transfected with blank plasmids, and those transfected with OSMR-siRNA + Tyr705 (*p* > 0.05). The aforementioned findings demonstrate that OSMR silencing inhibited while the activation of the JAK/STAT3 signaling pathway promoted the expression of inflammatory factors.

### Pathological reaction of CAU relieved and eosinophil number decreased after transfection with OSMR silencing

**Fig. 4:**
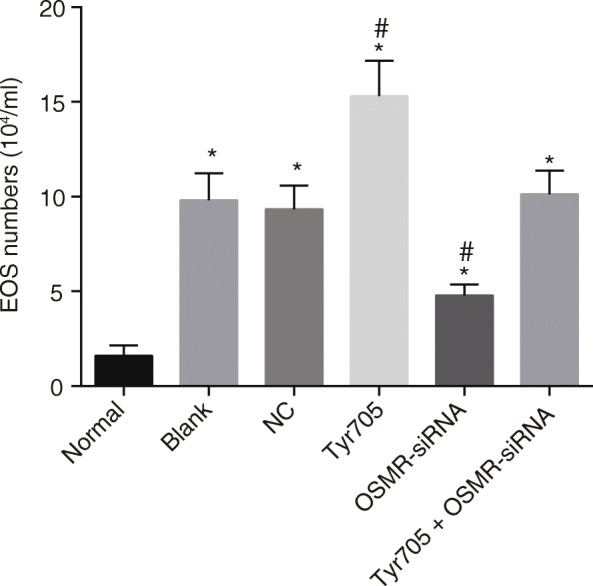
CAU pathological reaction relieved and eosinophils counting number decreased after CAU mice transfected with OSMR-siRNA (n = 12). Note: *, *p* < 0.05 compared with the normal group; #, *p* < 0.05 compared with the blank group; CAU, chronic autoimmune urticaria; OSMR, Oncostatin M receptor

As CAU is characterized by pruritus and flare reaction in skin with an increase in eosinophils, the pathological morphology in each group was observed under the microscope, and the number and duration of pruritus in CAU mice was accordingly analyzed and recorded. As shown by Table 2 and Fig. 4, the recorded number and duration of pruritus, and the eosinophils counting number were found to be increased in CAU mice (*p* < 0.05). Compared to the CAU mice with no transfection and transfected with blank plasmids, CAU mice tissues transfected with OSMR-siRNA exhibited significantly decreased number and duration of pruritus and eosinophils counting number, whereas the results were opposite in the CAU mice tissues transfected with Tyr705 (*p* < 0.05). Thereby, it can be concluded that OSMR silencing and JAK/STAT3 inhibition relieved CAU pathological reaction and decreased the eosinophils counting number.

**Table 2:** The number and duration of pruritus ratios in each group

Group	n	Number of pruritus (time)	Duration of pruritus (min)
Normal	12	0	0
Blank	12	57.15 ± 6.72^*^	28.00 ± 4.10^*^
NC	12	55.23 ± 4.38^*^	27.30 ± 3.90^*^
Tyr705	12	92.50 ± 7.80^*#^	40.50 ± 350^*#^
OSMR-siRNA	12	18.40 ± 5.10 ^*^	17.00 ± 3.35^*^
Tyr705 + OSMR-siRNA	12	56.38 ± 5.20^*#^	29.40 ± 3.40^*#^

^*^p < 0.05 compared with the normal group; ^#^*p* < 0.05 compared with the blank group; *NC* negative control, *OSMR* oncostatin M receptor

### Enhanced proliferation of epithelial cells after transfected with OSMR silencing and the JAK/STAT3 signaling pathway inhibition

**Fig. 5:**
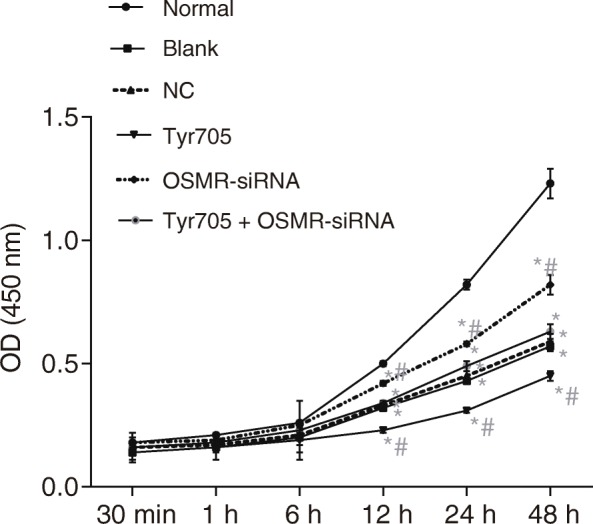
Epithelial cell proliferation ability inhibited after cells transfected with OSMR-siRNA. Note: the experiment repeated for 3 times; *, *p* < 0.05 compared with the normal group; #, *p* < 0.05 compared with the blank and NC groups; CAU, chronic autoimmune urticaria; OSMR, Oncostatin M receptor

Cell cycle and cell apoptosis are two essential elements for assessing cell growth and death, thus, flow analysis was performed in order to explore the alterations in cell cycle and cell apoptosis after different transfection. As showed by Fig. 5, cell proliferation was found to be decreased significantly with the decreasing condition remained for 6 h (*p* > 0.05). At transfection periods of 12, 24, and 48 h, cells transfected with OSMR-siRNA exhibited increases cell proliferation, while those transfected with Tyr705 had the lowest cell proliferation (*p* < 0.05). The findings reveal that OSMR silencing increased cell proliferation, while activation of the JAK/STAT3 signaling pathway inhibited cell proliferation.

**Fig. 6:**
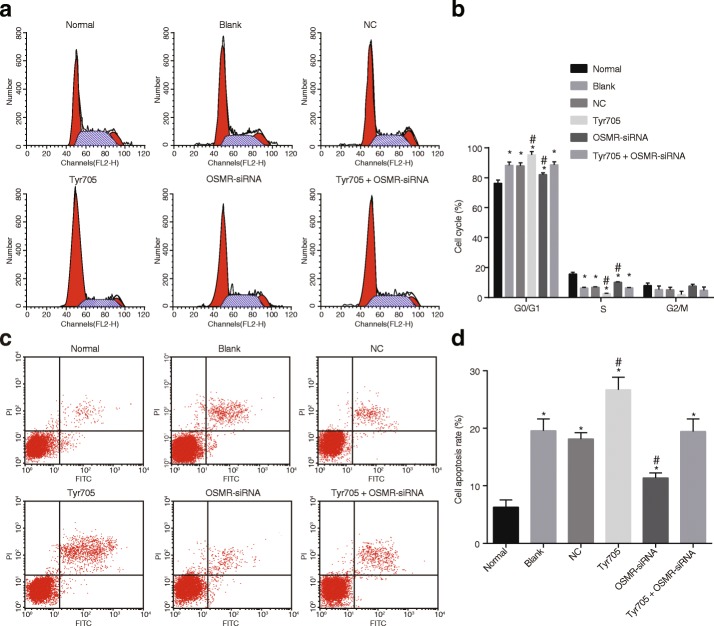
Shortened epithelial cell cycle and promoted cell apoptosis after cells transfected with OSMR-siRNA thus to inhibit CAU cell growth and promote their apoptosis. Note: **a** flow cytometry image revealed the epithelial cell cycle in different transfection groups; **b** histogram image displayed the epithelial cell cycle in different transfection groups; **c** flow cytometry image revealed the epithelial cell apoptosis in different transfection groups; **d** histogram image displayed the epithelial cell apoptosis in different transfection groups; the experiment repeated 3 times; *, *p* < 0.05 compared with the normal group; #, *p* < 0.05 compared with the blank and NC groups; CAU, chronic autoimmune urticaria; OSMR, Oncostatin M receptor

As results of the flow analysis demonstrate, compared with the normal group, epithelial cells had prolonged G0/G1 phases but diminished S phases, along with increased cell apoptosis (*p* < 0.05). Compared to the cells with no transfection or transfected with blank plasmids, cells transfected with OSMR-siRNA demonstrated diminished G0/G1 phases and prolonged S phases, whereas opposite results were observed in the cells transfected with Tyr705 (*p* < 0.05), and the results were not significantly different in cells transfected with Tyr705 + OSMR-siRNA (*p* > 0.05) (Fig. 6a, b). As for changes in cell apoptosis (Fig. 6c, d), decreased cell apoptosis was observed in cells transfected with OSMR-siRNA, whereas it was found to be increased in cells transfected with Tyr705, while there were no significant difference in cells transfected with Tyr705 + OSMR-siRNA (*p* > 0.05). The above results show that OSMR accelerated the apoptosis of epithelial cells by shortening the S phase and prolonging the G0/G1 phase of the cell cycle. Therefore, it can be concluded that OSMR silencing could shorten the G0/G1 phase, prolong the S phase of epithelial cells, thereby inhibiting epithelial cell growth, while down-regulation of the JAK/STAT3 signaling pathway promoted epithelial cell process.

### OSMR silencing inhibited the JAK/STAT3 signaling pathway thus suppressed CAU progression

**Fig. 7:**
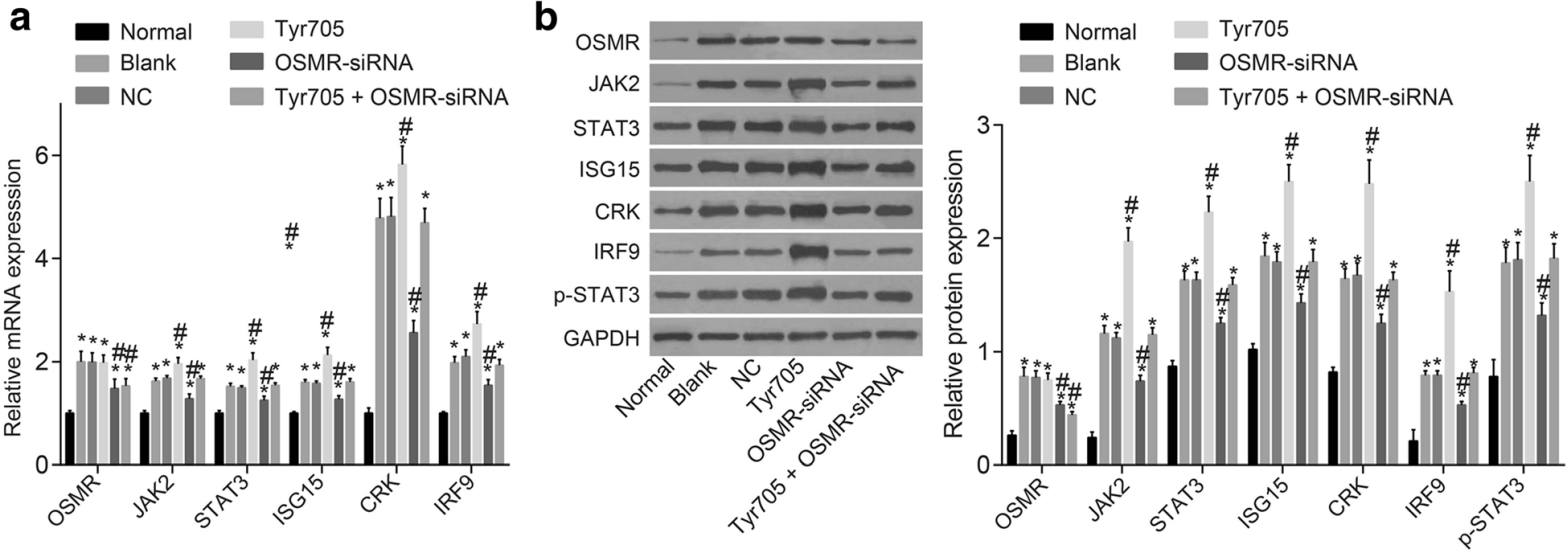
mRNA and protein expression of OSMR and the JAK/STAT3 signaling pathway related genes decreased after cells transfected with OSMR-siRNA and the JAK/STAT3 signaling pathway inhibition (n = 12). Note: **a** mRNA expression of OSMR, JAK2, STAT3, ISG15, CRK and IRF9 in epithelial cells and normal skin tissues; **b** protein expression of OSMR, JAK2, STAT3, ISG15, CRK and IRF9 in epithelial cells and normal skin tissues; *, *p* < 0.05 compared with the normal group; #, *p* < 0.05 compared with the blank and NC groups; CAU, chronic autoimmune urticaria; OSMR, Oncostatin M receptor; JAK2, janus kinase 2; STAT3, signal transducer and activator of transcription 3; ISG15, interferon-stimulated gene 15; CRK, CT10-regulated kinase; IRF9, interferon regulatory factor 9

Lastly, in order to assess the relationship between OSMR and the JAK/STAT3 signaling pathway in epithelial cells, RT-qPCR and Western blot assay were performed in order to explore the mRNA and protein expressions of OSMR and the JAK/STAT3 pathway related genes in epithelial cells with different transfection. As shown by Fig. 7a–c, compared to the normal cells, epithelial cells exhibited increased mRNA and protein expressions of OSMR and JAK/STAT3 signaling pathway related genes (*p* < 0.05). Compared with the epithelial cells transfected with blank vectors, epithelial cells transfected with OSMR-siRNA displayed decreased expressions of OSMR and JAK/STAT3 signaling pathway related genes (*p* < 0.05), while those transfected with Tyr705 exhibited elevated levels (*p* < 0.05). Interestingly, epithelial cells transfected with Tyr705 + OSMR-siRNA exhibited decreased OSMR and JAK/STAT3 compared to the epithelial cells transfected with Tyr705, indicating that OSMR could inhibit the JAK/STAT3 signaling pathway.

## Discussion

Chronic autoimmune urticaria (CAU), a commonly occurring disease, is accompanied by various symptoms including transient eruption of itchy, edematous swellings of the dermis, and erythematosus, lasting over a duration of 6 weeks (Wardhana, 2012). Recent study has shown that OSMR plays an important role in systemic lupus erythematosus, and further leads to the stimulation of various cytokines and inflammatory substances, such as IL-6 and IL-11 by activating the JAK/STAT pathway and the mitogen-activated protein kinase (MAPK) signaling pathways (Lin et al., 2014). The current study has shown that OSMR gene silencing can restrain the development of CAU, which can be achieved through blocking the JAK/STAT3 signaling pathway.

Initially, it was found that OSMR silencing inhibits the expression of the JAK/STAT3 signaling pathway related-genes (JAK2, STAT3, ISG15, CRK and IRF9). JAK2 is a non-receptor tyrosine kinase responsible for diverse cellular processes via stimulating cytoplasmic signaling cascades (Dawson et al., 2009). STAT3, another gene of interest in the current study, is a key member of the JAK/STAT signaling pathway (Yau et al., 2012). ISG15 is an ubiquitin-like protein whose conjugation is involved in the antiviral immune response and regulation of the JAK/STAT signaling pathway (Osiak et al., 2005; Hsiao et al., 2010). CRK belongs to Src homology-2 (SH2) and SH3 domain comprised of proteins that controls the coordinated combination of signaling complexes (Sriram et al., 2015). IRF9 is a crucial factor in the JAK/STAT signaling pathway that stimulates the antiproliferative function of IFN-α (Wu et al., 2017; Tsuno et al., 2009). A previous study found that both type I and type II OSMR activated JAK1, JAK2, and TYK2 receptor-associated tyrosine kinases (Auguste et al., 1997). Recently, OSM has been reported to stimulate ISG genes participating in antigen processing as well as presentation (Hergovits et al., 2017). The OSMR protein has been reported to be capable of heterodimerizing with IL-6 signal transducer (gp130) in order to produce type II OSMR, and when the receptor complexes were taken in, JAK could be activated, followed by further activation of STAT3 (Hong et al., 2011). Consistently, it has been revealed that the low expression of OSMRβ could decrease atherogenesis by inactivating the JAK2/STAT3 signaling pathway in macrophages (Zhang et al., 2017). The aforementioned findings and evidence suggest that OSMR gene silencing could suppress the JAK/STAT3 signaling pathway.

Additionally, the current findings demonstrate that the contents of IL-1, IL-6, IFN-γ and IgE in serum of mice in the OSMR-siRNA group were significantly reduced, indicating that OSMR gene silencing suppressed the autoimmunity of CAU by blocking the JAK/STAT3 signaling pathway. Notable, it was reported that regulation of IFN-γ-secreting T helper 1 cells could inhibit autoimmunity and immunopathology (Cope et al., 2011). Previous studies have demonstrated that OSMR could increase IL-1 and TNF activity in synovial fibroblasts, which was consistent with the results of the current study (Le Goff et al., 2014). Interestingly, it was reported that inhibition of OSMR results in suppressed IL-31, which was highly expressed in the skin of patients with chronic spontaneous urticaria and was released from isolated basophils accompanied with anti-IgE activation (Raap et al., 2017). Moreover, it has been reported that inactivation of the JAK/STAT pathway could help in inhibiting the expression of ICAM-1 induced by IFN-γ in HaCaT human keratinocytes (Sung & Kim, 2013). The activated JAK/STAT signaling pathway could stimulate IFNs in order to exert an innate immune response (Cheng et al., 2014). In addition, STAT3 mutations are associated with autosomal dominant-hyper-IgE syndromes (AD-HIES) and this association might allow differentiation of AD-HIES from disorders correlated with elevated serum IgE levels (Schimke et al., 2010).

Consequently, it was revealed that OSMR gene silencing can obstruct the development of CAU by inhibiting the JAK/STAT3 signaling pathway, as increased proliferation, migration and decreased apoptosis of epithelial cells were observed in the OSMR-siRNA group. OSM, is a cytokine capable of modulating cell survival and proliferation, and the over-expression of OSM could result in transdifferentiation of epithelial-myofibroblast (Elbjeirami et al., 2010). In line with the findings of the current study, it was reported that over-expression of OSM in tubular epithelial cells might aggravate mucosal epithelial barrier dysfunction (Pothoven et al., 2015). The JAK/STAT is capable of modulating signaling cascades exerting great effects on proliferation, differentiation, development as well as immune responses (Kim et al., 2011). Additionally, it was reported that acute nitrogen dioxide exposure enhances airway inflammation that both humoral immunity and cellular immunity reaction via modulating Th1/Th2 differentiation and activating the JAK/STAT signaling pathway (Ji et al., 2015). Therefore, it can be hypothesized that OSMR gene silencing regulates proliferation, migration as well as apoptosis of epithelial cells in CAU by inhibiting the JAK/STAT signaling pathway.

## Conclusion

The current study suggested that OSMR gene are highly expressed in human CAU skin tissues, and cause the up-regulation of the JAK/STAT3 signaling pathway-related genes. Additionally, it was demonstrated that OSMR gene silencing significantly decreases the content of inflammatory factors, the number of eosinophils, and reduces the mRNA and protein expressions of JAK/STAT3 signaling pathway-related genes, enhances cell proliferation, migration and inhibits apoptosis of epithelial cells. Thereby, it can be concluded that OSMR gene silencing inhibits autoimmunity in CAU mouse models by inactivating the JAK/STAT3 signaling pathway. These findings may open novel avenues for future CAU therapies and to ultimately, raise the quality of life of CAU patients. However, the limited sample size of the current study remains to be a limitation. Thus, further studies are warranted in order to better the understanding of specific mechanisms.
